# Waking Up Blind in the ICU: A Case Report of Ischemic Optic Neuropathy in a Burn Patient

**DOI:** 10.7759/cureus.5437

**Published:** 2019-08-20

**Authors:** Luis Quiroga, Mohammed Asif, Tomer Lagziel, Julie Caffrey

**Affiliations:** 1 Surgery, Burn Center, The Johns Hopkins University School of Medicine, Baltimore, USA; 2 Surgery, The Johns Hopkins University School of Medicine, Baltimore, USA; 3 Medicine, Burn Center, The Johns Hopkins University School of Medicine, Baltimore, USA

**Keywords:** blindness, ischemic optic neuropathies, non-arteritic ischemic optic neuropathy, burn complications, ion, naion, neuro-ophthalmology

## Abstract

The objective of this report is to analyze and summarize the current literature of ischemic optic neuropathy (ION), a rare complication in severe burn and trauma victims, while presenting an urban burn center's experience with the condition. This is an unfortunate condition and this report will raise awareness to a potential complication in the burn patient population as well as in critically ill patients in other settings.

We present the case of a 27-year-old healthy male patient admitted to our Burn Center with 85% total body surface area (TBSA) full-thickness burns sustained in a house fire. The patient had a complicated hospital course but improved over time and was weaned off of prolonged ventilation and sedation. Subsequently, he complained of bilateral blindness. A fundoscopic examination demonstrated bilateral pale optic nerves with sparing of the remaining peripheral retina consistent with ION. The patient suffered complete bilateral vision loss. He had multiple factors that could have instigated the development of ION, including several episodes of septicemia, hypovolemic shock and severe adult respiratory distress syndrome (ARDS) with refractory hypoxemia requiring a prolong ventilation support and vasopressor therapy.

Due to the advancement of the treatment of acute burns, the survival rate of patients that once would have succumbed to their burn injury, is increasing. With these new achievements, we are facing new challenges and complications. ION has a significant impact on the quality of the patient’s life. The early diagnosis will not necessarily translate into a benefit for these patients as no treatment has been proven successful. Extensive retrospective and prospective studies are necessary to identify and treat this patient population.

## Introduction

Optic nerve damage resulting from decreased blood flow is known as Ischemic Optic Neuropathy (ION). ION is classified into two types: anterior (AION) and posterior (PION) [1]. The AION can be the result of increased optic pressure and edema as well as decreased blood flow to the anterior optic nerve. It is hypothesized that the development of this compartment syndrome in the anterior portion of the optic nerve is the critical pathway for the occurrence of this complication [2,3]. Ischemic optic neuropathy (ION) is an uncommon complication with significant and devastating implications for burns patients and their families following severe burns and trauma due to high levels of blood loss [4]. It is not a direct consequence of the burn injury, but rather the result of multiple factors in a process poorly understood. Here, we discuss our experience in the light of our current knowledge of this pathology.

## Case presentation

A 27-year-old male with no previous medical history was admitted to the Burn Intensive Care Unit (BICU) following a house fire. The patient was conscious on arrival at the hospital and interacting with 85% TBSA full-thickness flame burns to his face, head, neck, entire torso, groin, perineum, buttocks, lower back and circumferential burns to the entirety of all four extremities, and with concomitant inhalation injury. He did not receive any fluids in the field or during transport, as the paramedic could not obtain IV access. Intubation was performed in the ED for airway protection. Bronchoscopy revealed evidence of thermal injury at the carina and main bronchi consistent with inhalation injury. Escharotomies were performed. The patient was resuscitated in the first 24 hours with a total of 42.5 liters (L) of crystalloids (Parkland Formula 4cc x125kg x 85% TBSA=42,500mL/24 hours). He did not require any vasopressors during this time. 

An ophthalmology consultation was obtained. Due to the extreme condition of the patient, a full 8-point ophthalmologic exam could not be completed. Visual acuity, visual fields, and extraocular movement could not be determined. Tonometry revealed 14mmHg in the right eye and 13mmHg in the left. Pupil reactivity tests confirmed no afferent pupillary defects. An ophthalmologic exam also revealed exposure keratopathy of both eyes, which means that the corneas were damaged as a result of prolonged exposure to the outside. It also revealed a severe corneal epithelial defect of the left eye. However, the funduscopic exam was normal. A Prokera lens was placed, and topical antimicrobials and moisture chambers were applied to the eyes.

**Figure 1:**
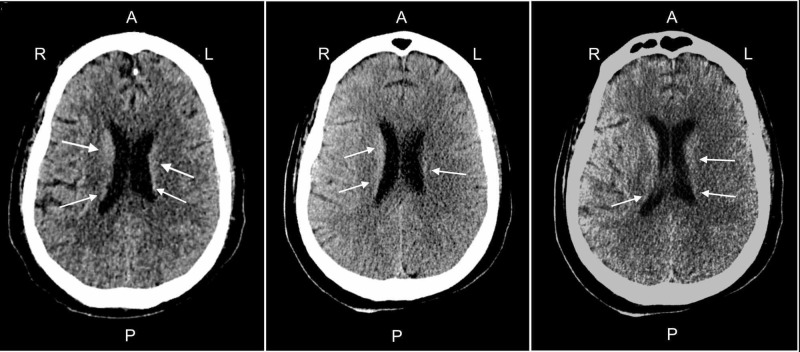
Head CT without contrast from different angles. Blood pooling in the anterior aspect of the lateral ventricles into the occipital horns bilaterally. A - Anterior P - Posterior R - Right L - Left

During his hospitalization, the patient’s course was complicated by severe ARDS with refractory hypoxemia requiring prone positioning and vecuronium for paralysis. Also, he developed several episodes of sepsis with multi-organ failure and septic shock, requiring vasopressor support. Secondary to compartment syndrome, he required bilateral below-knee amputations. Neurosurgery and neurology were consulted for pupillary anisocoria and pupil non-reactivity. A CT scan of the head demonstrated a very small occipital horn intraventricular hemorrhage and possible cortical subarachnoid hemorrhage [Figure 1]. No intervention was indicated. 

When the patient’s mental status improved, he reported that he was unable to see. Neuro-ophthalmology performed a funduscopic examination that demonstrated bilateral pale optic nerves with sparing of the remaining peripheral retina, suggestive of a chronic process vs. acute change. Given his periods of hypotension, anemia, and multiple surgeries, the patient’s findings were consistent with ischemic optic neuropathy.

The patient was discharged from the hospital after further recovery, at this time reporting complete bilateral vision loss with only recovery of mild perception to light.

## Discussion

The pathophysiology of Ischemic optic neuropathy (ION) is a highly complex process that involves multiple factors. It is a prevalent cause of blindness among the elderly after glaucoma and optic neuritis, but a rare and devastating complication in burn patients. The majority of ION is non-arteritic [1]. The annual incidence is estimated at 2.3 to 10.2 cases per 100,000 in persons 50 years of age or older. Men and women are equally affected, and the vast majority (>95%) are Caucasian. It is accepted that small vessel circulatory insufficiency of the optic nerve head is the most likely cause. Nevertheless, ION is a distinct process in the burn patient population, as well as patients with severe trauma. Unlike the typical ION patient, these patients are mainly young and previously healthy with no evidence of cardiovascular disease or other associated risk factors.

A review of the scientific literature revealed that the reported cases in burn and trauma patients share common elements sufficient to infer a predictive pathophysiology model. These patients commonly share the presence of extensive injuries requiring a significant amount of fluid resuscitation in the first 24 hours (usually more than 20 L in the first 24hr “massive fluid resuscitation”) to prevent shock. Burned patients develop a well-known systemic inflammatory response (SIRS), associated with significant capillary leak and anasarca. The combination of these two elements (copious amounts of fluids and the capillary leak) predispose these patients to the development of compartment syndrome [2,3].

Additionally, all the reported burned patients who suffered ION develop severe episodes of hypoxemia and global hypoperfusion during their hospitalization. Frequent events in these reported patients include ARDS requiring high levels of oxygen, acute anemia requiring a blood transfusion, and several episodes of sepsis requiring multiple pressors. Several publications have identified the association between hemorrhagic shock and ION outside the burn population [4-7]. In our case, the patient had an episode of hemorrhagic shock when he bled from his donor sites after becoming refractory to epinephrine soaks. He later required topical Tranexamic acid (TXA) for his donor sites to prevent bleeding.

It is important to note that not all the patients with these variables end up developing ION. Cullinane et al. reviewed 350 trauma cases which required more than 20L of fluid resuscitation in the first 24hr (massive volume resuscitation) and found that only nine patients (2.6%) developed ION [8]. Nevertheless, the number of ION in burned patients is less well-defined, as a review of national data has not yet been performed. A study like this could reveal similar findings to that of Cullinane et al. in the Trauma population. To our knowledge, this is the 6th case reported in burn patients [9-11]. Consequently, this relatively small number of cases with ION indicates an idiosyncratic factor in these patients. It has been suggested that at least one of these factors is the presence of a small cup/macula ratio (a ratio of the optic to the optic disc - used to diagnose glaucoma). This anatomical feature predisposes the patient to compartment syndrome of the eye [12]. Interestingly, in Cullinane’s report, 56% of the patients who developed ION (5 patients) suffered monocular blindness (left eye) whereas four patients (44%) had bilateral blindness supporting the idea of an anatomical difference variable.

It is thought that the development of compartment syndrome in the anterior portion of the optic nerve is one of the possible causes of this complication. The compartment syndrome occurs in a fixed area, most likely where the optic nerve passes through the lamina cribosa. This compartment syndrome is caused by the edematous nerve fibers within the optic nerve and a small scleral canal at the lamina cribosa. This increased pressure in the compartment causes a venous outflow obstruction and critical venous hypertension followed by secondary arterial hypoperfusion and ultimately infarction of the anterior optic nerve. This mechanism also explains why prone positioning (either for severe and refractory global hypoxemia in ARDS or surgery positioning) might contribute to the development of AION in this patient by causing venous hypertension.

The recognition of this complication is usually delayed due to prolonged ventilatory support and sedation. Initial eye exam during the first hours of admission might reveal corneal defects secondary to the burns, but normal retina and eye light reflex. When the patient can communicate, they usually report painless vision loss. The diagnosis is primarily clinical, and the eye exam confirms the presence of an afferent pupillary defect and optic disc pallor and edema with sparing of the remaining peripheral retina on fundoscopy [13]. A crucial finding on examination is the presence of a small cup-to-disc ratio (disc at risk), meaning a crowded optic-nerve head with a small physiological cup [12,14,15]. On the other hand, the posterior ION (PION) has no well-known structural risk factors.

There is no proven therapy to impact or reverse the outcomes of this complication. In 1989, Sergott and Savino proposed that optic nerve decompression surgery (ONDS) might improve vision in the patient with a progressive form of NAION (not secondary to trauma or burn) [16]. However, the Ischemic Optic Neuropathy Decompression Trial (IONDT), a singled-masked, multicenter randomized controlled clinical trial sponsored by the National Eye Institute, concluded that ONDS is not safe, and in fact might be harmful; hence it was abandoned [17].

Evans and Sullivan recommend routinely using tonometry to measure the intraocular pressure in patients with severe burns and orbital congestion in the setting of large amounts of intravenous fluids [18,19]. Sullivan et al. reviewed 13 consecutive patients with TBSA of more than 25 % of which only 5 of 13 had intraocular pressure (IOP) higher than 30 mmHg and required prophylactic lateral canthotomies [19]. No prospective studies have been done to prove any benefit of this procedure in preventing ION in burn patients.

In summary, our patient had multiple factors that predisposed him to develop ION. It is essential to have a high index suspicion for early recognition of ION, especially in patients with extensive burns. However, extensive retrospective and prospective studies remain a necessity to understand this pathology and potentially develop early interventions that could save these patients from blindness and debilitation.

## Conclusions

Further extensive retrospective and prospective studies remain necessary to understand more about burn-induced ION and to develop potential treatment and prevention methods. Early diagnosis is difficult, and although it may not benefit these patients, it will help with family discussion of prognosis and expectation. Clinical suspicion should be followed by fundoscopy. Prevention is the best option available at this moment. Judicious fluid resuscitation and minimizing hypoxemia and hypotension periods as well as selective pronation of patient with refractory ARDS remains the primary option to prevent ION.
